# Is Testicular Torsion a Real Problem in Pediatric Patients With Cryptorchidism?

**DOI:** 10.3389/fped.2020.575741

**Published:** 2021-01-12

**Authors:** Marta Diana Komarowska, Alicja Pawelczyk, Ewa Matuszczak, Wojciech Dębek, Adam Hermanowicz

**Affiliations:** Department of Pediatric Surgery and Urology, Medical University of Bialystok, Bialystok, Poland

**Keywords:** undescended testis, testicular torsion, pediatric population, cryptorchid testes, boys

## Abstract

**Purpose:** To present management and outcomes of patients with cryptorchidism suffering from testicular torsion.

**Methods:** This is a retrospective review of pediatric patients with torsion of undescended testes, who were treated between 2009 and 2019. We recorded: the age, symptoms, duration of torsion, results of physical examination, surgery findings, and additional treatment. Additionally, an extensive online literature search, from 2015–2020, was performed to identify other similar case series.

**Results:** We identified 9 boys with torsion of the undescended gonad, which represented 4.7% of all 192 boys with testicular torsion. The mean age of boys with torsion of undescended testis was 8.7 years. The mean duration of symptoms in our study group was long and it was up to 28.5 h. All patients had inguinal canal exploration. In eight cases, testicular necrosis and primary orchidectomy was performed. Furthermore, 45 cases were identified in the literature and reviewed.

**Conclusion:** According to clinical experience and available studies, torsion of male undescended gonads is a comparatively rare condition. Nevertheless, diagnosis is still delayed and connected with inevitable orchidectomy.

## Introduction

Acute scrotum is a medical emergency. In the pediatric population, the most common causes of this pathology are usually: hydatid or testicular torsion and epididymo-orchitis (1, 2). The main symptoms are most commonly: scrotal pain, swelling and redness of the scrotum. Nevertheless, testicular torsion is the most serious cause of acute scrotum and may result in the loss of the testicle. Testicular torsion could be extra- or intravaginal. Usually extravaginal is typical for the neonatal period and includes the testicle, epididymis and tunica vaginalis. Contrary to extravaginal, intravaginal torsion is connected with a bell-clapper deformity. In this abnormality, the mesorchium terminates early and the testis is free floating in the tunica vaginalis.

Cryptorchidism is the most common genital disorder in males (3), and generally should be diagnosed and treated until 18th month of life (4). The consequences of an undescended testicle are higher risk of testicular cancer (5) and impaired fertility (6). Moreover, cryptorchidism is connected with a higher risk of gonadal torsion (7–9). Because of the risk of testicular necrosis, torsion of the gonad is the most urgent disorder and surgical emergency. Therefore, prompt diagnosis and urgent surgical treatment are crucial.

Testicular torsion can usually be diagnosed based on a careful physical examination and appropriate Color-Doppler Ultrasound (CDU). However, one should consider the fact that physiologically in prepubertal boys and neonates intratesticular blood flow is reduced, which may result in false-positive ultrasound results. Correct interpretation seems to be more difficult in the case of incorrect placement of the gonads (undescended testis –inguinal and especially abdominal undescended testis). Approximately 80% of undescended testicles are palpable (3) and located in the inguinal canal. Physical examination is a basic technique in the diagnosis of cryptorchidism.

The purpose of this study is to present management and treatment outcome in patients with cryptorchidism suffering from testicular torsion.

## Materials and Methods

In this retrospective study, we analyzed all boys with testicular torsion, who were operated on in the Pediatric Surgery and Urology Department of the Medical University of Bialystok (Poland) between 2009 and 2019. We recorded: age, symptoms, duration of torsion, results of physical examination, surgery findings and additional treatment. Patients with torsion of undescended gonads were selected and included into the study. Additionally, we compared our case series with the data from the literature, including similar case series, excluding case reports and one study with unavailable data (10).

### Statistical Analysis

Statistical analysis of the data was done using SPSS 21.0 for Windows (SPSS Inc.Chicago, IL, USA). The collected data were processed using the methods of descriptive and analytical statistics. The quantitative data were expressed as mean value ± standard deviation (MV ± SEM). Significance of the difference was obtained using Student *t*-test or non-parametric Mann-Whitney test, Fisher's contingency tables and Hi-square test. Statistical hypotheses were tested at the level of statistical significance of *p* < 0.05.

## Results

Over the last 10 years (from 2009 to 2019), 192 boys with testicular torsion were operated on in our Department. In this group, we identified nine boys with torsion of the undescended gonad, which represented 4.7% of all boys with testicular torsion. Six patients had torsion of the left testicle (66.7%).

The described nine patients with torsion of undescended gonads accounted for 0.95% among 860 boys operated on in a 10-year period because of cryptorchidism (congenital and ascending gonads).

The mean age of boys with torsion of undescended testis was 8.7 years (range 6 months−14 years).

Among our patients, six were in general good health, three other subjects suffered from cerebral palsy (one of them had also epilepsy).

The most common complaints in all patients were local symptoms: painful inguinal swelling and redness of the groin. Additionally, we noticed general manifestations like abdominal pain, vomiting and fever.

The mean duration of symptoms in our study group was 28.5 h (range 6 h−4 days). Only one patient was admitted to the hospital during the first 6 h from the onset of symptoms. In all cases, a diagnosis of cryptorchidism was made. In three patients bilateral cryptorchidism was diagnosed at the age of 10, 11, and 13 years, respectively. Interestingly, these 3 boys with cerebral palsy had their diagnosis of cryptorchidism made a few years earlier. Nevertheless, caregivers did not decide to have their children operated on.

Urgent Color-Doppler Ultrasound was performed before operation in all boys. In two cases (22.2%), proper diagnosis had been made. In one patient, preoperative CDU suggested enlarged lymph node. In the CDU of three boys, inflammation of inguinal testis and epididymis was suspected. In two cases, a diagnosis of appendage torsion was made, and in 1 case, after CDU examination, suspicion of incarcerated inguinal hernia was made.

**Figure 1:**
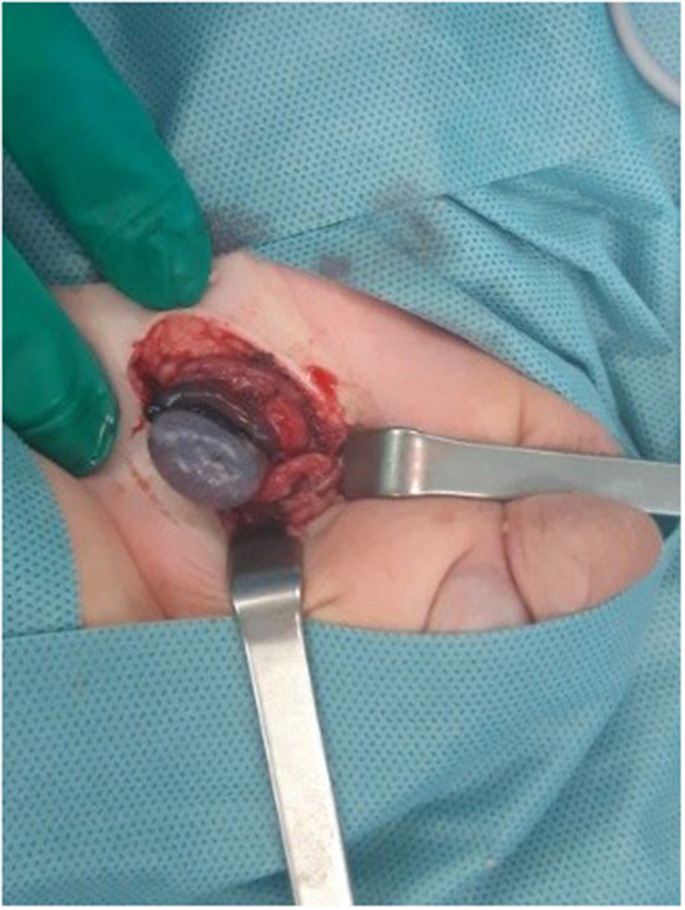
Extravaginal inguinal testicular torsion in a 6 month-old boy.

Regardless of the results of the CDU examination, all patients with the symptoms of torsion underwent urgent surgical inguinal region exploration. In one case of a 6-month neonate, extravaginal torsion was detected. In other cases, the intravaginal form of torsion was found. In eight cases of boys with testicular torsion, we found testicular necrosis, and primary orchidectomy was performed (Figure 1). In one case, during surgery we noticed proper reperfusion of the gonad and orchiopexy was performed. Unfortunately, this patient did not return for follow-up. In all cases, fixation of the other testis was done. In three patients with bilateral cryptorchidism orchiopexy was performed during the same procedure. In the postoperative period, all boys were given intravenous antibiotics (cefuroxime-7 patients, amoxicillin—clavulonate-2 patients). We did not notice any postoperative complications, like wound infections or fever (Table 1).

**Table 1:** Case series presentation of children with testicular torsion.

**Patient**	**Age (month)**	**Side**	**Duration (hours)**	**Symptoms**	**Bilateral cryptorchidism**	**Surgery**	**Comorbidity**	**Follow-up: Normal/Atrophy/Hypotrophy/NA**
1	120	L	48	Inguinal pain, tender mass	yes	Necrosis–orchidectomy	Cerebral palsy Epilepsy	
2	120	R	48	Painful inguinal swelling Redness, tender mass	no	Necrosis–orchidectomy	Healthy	
3	156	L	9	Painful inguinal swelling	yes	Necrosis–orchidectomy	Cerebral palasy	
4	6	L	96	Painful inguinal swelling Fever	no	Necrosis–orchidectomy	Healthy	
5	24	L	6	Painful inguinal swelling, inguinal redness Abdominal pain Vomiting	no	Necrosis–orchidectomy	Healthy	
6	60	R	10	Painful inguinal tender mass	no	Orchidopexy	Healthy	NA
7	132	L	12	Painful inguinal tender mass	yes	Necrosis–orchidectomy	Healthy	
8	156	R	24	Painful inguinal swelling, inguinal redness	no	Necrosis–orchidectomy	Cerebral palsy	
9	168	L	96	Painful inguinal swelling, inguinal redness	no	Necrosis–orchidectomy	Healthy	

*NA, not available*.

### Analysis of the Literature Review

Analyzing the data from previous publications of other authors we did a statistical analysis of our case series of testicular torsion in cryptorchidism and the data from the literature (Table 2) (11–15). Apart from our nine cases we found 45 pediatric cases in five previous publications of different authors, dating from 2015–2020. All studies deal with similar small number of patients, from 6–13 participants, with no statistically significant difference among them. The clinical complains were similar, too.

**Table 2:** Literature review of case series analysis of testicular torsion in cryptorchidism.

**Study**	**No of Patients**	**Age (month) (MV ± SEM) *p***	**Side R/L (%)**	**Duration of symptoms (hour) (MV ± SEM) *p***	**Bilateral cryptorchidism(%) *p***	**Surgery: Orchidectomy/Orchidopexy (%) *p***	**Co morb idity (%)**	**Follow-up (%)**			
								**Normal testicle**	**Atrophy**	**Hypotrophy**	**NA**
1. Pogorelić et al. (11)	8	0,2–192 (141 ± 22,16) ***p*** **=** **0.0562**	5/3 (62,5/37,5)	4–60 (28,13 ± 8,65) *p* = 1	0	4/4 (50/50) *p* = 0.7032/*p* = 0.3233	0	3 (38)	1 (13)	0	0
2. Sener et al. (12)	13	8–70 (29 ± 5,61) ***p*** **=** **0.0383**	5/8 (38,5/61,5)	4–84 (16,54 ± 5,89) *p* = 0.7543	0	4/9 (31/69) *p* = 0.2818/*p* =0.1140	0	0	2 (15)	0	7 (54)
3. Naouar, Braiek, and El Kamel, (13)	12	12-156 (92 ± 12.48) *p* = 0.1499	6/6 (50/50)	3-48 (14 ± 3,62) *p* = 1	3 (25)	6/6 (50/50) *p* = 0.4998/*p* = 0.3642	0	1 (8)	2 (17)	3 (25)	0
4. Ito et al. (14)	6	12–192 (122 ± 32,36) *p* = 0.3359	3/3 (50/50)	1-20 (64,17 ± 17,03) *p* = 1	5 (83)	5/1 (83/17) *p* = 1/1	6 (100)	0	1 (17)	0	0
5. Deng et al. (15)	6	0,4–132 (60,17 ± 23,10) *p* = 0.1442	2/4 (33/67)	12–22 (20,5 ± 4,05) ***p*** **<** **0.0001**	0	3/3 (50/50) *p* = 0.6828/*p* = 0.3034	0	3 (50)	0	0	0
6. Our case series	9	6–168 (104,7 ± 19,98)	3/6 (33/67)	6–96 (38,78 ± 12,04)	3 (33)	8/1 (89/11)	3 (33)	0	0	0	1 (11)
p	0.4922–1		0.5310		0.4–1	0.2818-1/0.1140-1	0.40	0.3–1	1	/	0.2–1

*MV ± SEM, mean value ± standard deviation; NA, not available*.

In two studies, participants were much younger: in study *No 1. Pogorelic et al*. (11), the youngest patient was only 7 days old (*p* = 0.0562), with wider range of participants' age; but in *No 2. Sener et al*. (12), the oldest patient was only 5 years and 10 months old (*p* = 0.0562), with shorter range of participants' age. Both studies showed a statistically significant difference compared to our series of patients. In all other studies the range of participants‘ age was similar: between several months to 16 years, with no statistically significant difference to our series of patients (Table 2).

Only one study, *No 1. Pogorelic et al*. (11), was dominated by torsion of the right testicle (5/3; 62,5/37,5%), in two studies, *No 3. Naouar et al*. (13), and *No 4. Ito et al*. (14), the distribution right/left side was equal (50/50%), while in other two studies the affectation of the left testicle was dominant, in 67% of cases, as in our series, but in total, with no statistically significant difference (Table 2).

In terms of duration of symptoms, in one study, *No 5. Deng et al*. (15), the range was only 10 h (12–22; *p* < 0.0001), which represents a statistically significant difference in relation to our series of patients. Similar to our study, the duration of symptoms in other studies is between several hours to several days, with no statistically significant difference among them (Table 2).

Bilateral cryptorchidism was found in two more studies in the literature, represented in 25–83% of patients, with no statistically significant difference in relation to our series of patients (33%).

As far as treatment is concerned, in three studies: *No 1. Pogorelic et al*. (11), *No 3. Naouar et al*. (13), and *No 5. Deng et al*. (15), there was an equal number of orchidectomy vs. orchidopexy (50/50%). In the study *No 4. Ito et al*. (14), there was very small number of saved testicles, in 17% of cases and in only one study, *No 2. Sener et al*. (12), one with youngest patients, the orchidopexy was done in 69% of cases, which is still, in total, with no statistically significant difference to our series of patients.

Only one study describes comorbidities in patients with torsion of undescended testicle. In *No 4. Ito et al*. (14), all 6 (100%) patients suffer from comorbidity: four from cerebral palsy and two from neuromuscular disease, with no statistically significant difference in relation to our series of patients.

Testicular salvage rate was encountered in all reviewed studies, with different outcome of affected testicles. In two studies, *No 1. Pogorelic et al*. (11), and *No 5. Deng et al*. (15), normal testicle in the later follow-up was found in 3 (38%) and in 3 (50%) cases, respectively. *No 3. Naouar et al*. (13), report only one case (8%) of normal testicle. Atrophy and hypotrophy of the affected testicles, are reported in four studies: in *No 1. Pogorelic et al*. (11), 1 (13%); *No 2. Sener et al*. (12), 2 (15%); *No 3. Naouar et al*. (13), 5 (42%) and in No *4. Ito et al*. (14), 1 (17%) of all cases, with no statistically significant difference among them, bearing in mind that such data were not available in our study for only one salvaged testicle (11%) and in *No 2. Sener et al*. (12), for 7 (54%) salvaged testicles.

## Discussion

The first case of testicular torsion was described in 1840, by Delasiauve (16). It was a teenager with cryptorchidism, and orchidectomy of necrotic testis was performed. Nevertheless, the incidence of torsion in cryptorchid patients is still unknown. Generally in the literature, a few descriptions of a series of cases can be found (9, 17–19). There are no randomized trials with unambiguous guidelines. During 16 years, Naouar et al. (13) found 13 cases of torsion of undescended testicle, in one adult and 12 children. Similarly, other authors (10) presented a two-center review of 11 patients with torsion of undescended testicle, who accounted for 9.7% of all boys with testicular torsion. In our study, 4.7% of patients with testicular torsion suffered from cryptorchidism.

The mechanism of testicular torsion of the undescended testis is not well-understood (18). There are some theories about the pathophysiology of torsion of the undescended testis. Incorrect spasm or contraction of cremasteric muscle is one of them (13, 18). Additionally, in children with cerebral palsy contracture of the hips could be a potential risk factor (20). A recent study suggested that the prevalence of cryptorchidism in patients with cerebral palsy is approximately 10-fold greater than in the general population (21). It is also strongly associated with spastic quadriplegia. In our series, three patients suffered from cerebral palsy. Another risk factor is an increased size of the gonad in patients with testicular tumors (8). We did not find any cases of tumor in our patients. On the other hand, similarly to scrotal torsion, we found that the extravaginal type was recognized only in a 6-month-old neonate. There are two peaks in the incidence of testicular torsion in the pediatric population: during the 1st year of life and between 12 and 18 year of life (22, 23). In our patients, we also observed the same age - dependency.

Clinical signs are less obvious than usually. We know that acute scrotal pain, especially connected with additional clinical signs is an emergency and requires rapid medical consultation. Pain or swelling in the inguinal canal are not so alarming. In this kind of patient we should differentiate between incarcerated hernia, appendicitis, lymphadenitis, renal colic or groin injury (24). As shown in our study, patients suffer from non-specific complaints like fever, vomiting or abdominal pain. It should be emphasized that a meticulous physical examination (including inguinal canal and scrotum area) is crucial and increases the chance of saving the testicle.

Imaging tests are useful for a proper diagnosis. Ultrasound examination is a safe, accessible and fast tool to make a proper differential diagnosis. In different reports, the sensitivity of color Doppler ultrasound ranged from 69.2 to 100% (2, 25, 26), although it could be challenging and depends on the experience of the physician. In our study, only in 22.2% of the cases the correct ultrasound diagnosis has been made. Regardless of the results of the CDU, all patients with cryptorchidism and suspicion of testicular torsion were qualified for urgent surgical intervention.

Testicular salvage is possible when a surgical intervention is performed within the first 6–8 h from the beginning of the symptoms (27, 28). According to a systematic review (29), when the spermatic cord is untwisted within 6 h, 97.2% of the testis survives. In our series, the time to surgical intervention was much longer. It seems that one of the reasons is low awareness of the caregivers. Friedman et al. showed that only 34% of parents are aware of this condition (30). According to the literature, one-third of cases of scrotal torsion ended with orchidectomy (31, 32). In our series up to 89% of subjects were treated with orchidectomy. This was due to the fact that the caregivers underestimated the children's symptoms. In two cases because of lack of awareness, caregivers delayed the visit to the doctor for 4 days.

Karl & Haid (10) in their study report testicular salvage rate of only 18%. Presentation of the patients with inguinal pathology, especially of very young age, might negatively influence testicular salvage rate. On contrary, in the study No 2. Sener et al., (12) the one with youngest patients in our literature review, the orchidopexy was done in 69% of cases.

The total testicular salvage rate among all 54 compared cases in our study is 7 (13%) and it is the lowest score, while failed testicles are revealed in 9 (17%), or not available data in the rest of 8 (15%) cases, with no statistically significant difference among them.

What seems to be important, our case series also shows that treatment of patients with cerebral palsy with coexisting cryptorchidism is an unsolved ethical dilemma (33). According to the Nordic recommendation, orchiopexy should be performed between the 6th and the 18th month of life (34). Nevertheless, there is an ethical discussion about patients with neurological disorders. Usually, caregivers' wishes regarding treatment options are taken into account. It appears that especially non-palpable and high positioned testes are associated with a potentially higher risk of the negative consequences of this disorder (35), which confirms Allin et al., reporting that atrophy and complication rates do not appear different between early and delayed orchidopexy (36). Therefore, the positioning of the gonad in the subcutaneous area in the inguinal canal enables further control. Furthermore, it is highly recommended to combine orchiopexy with other surgical procedures (orthopedic, etc.). We should also bear in mind the possibility of intra-abdominal testicular torsion (11, 37, 38). Usually, the most common symptom is abdominal pain. In the literature, the reports of cases of testicular torsion of the intra-abdominal seminoma can be found (39, 40).

The main limitation of this study is typical for the case series: retrospective analysis and small number of cases, which can lead to the danger of over-interpretation and the lack of generalization. On the other hand the strength of our survey is, that this is probably one of the biggest series coming from one center. Further studies taking into account other national Pediatric Surgery Departments are necessary. Such multicenter studies would probably significantly increase the study group and provide more data.

## Conclusion

According to clinical experience and the available studies, torsion of the male undescended gonad is a comparatively rare condition. Nevertheless, male patients with inguinal swelling and tenderness should be carefully and urgently examined. All patients need surgical/urological consultation. As it is presented in our research, as well as in the reviewed articles from the literature, diagnosis is still delayed and connected with the inevitable orchidectomy, and with negative influence to the testicular salvage rate. Both physicians and caregivers should be aware of possible torsion of the inguinal testis to increase the possibility of saving the gonad.

Our study indicate that the risk for undescended testis torsion really exists, however it is not very high. The diagnosis is usually delayed due to unspecific symptoms and signs with fatal consequences.

## Data Availability Statement

The original contributions generated for this study are included in the article/supplementary materials, further inquiries can be directed to the corresponding author/s.

## Ethics Statement

The studies involving human participants were reviewed and approved by Ethics Committee of Medical University of Bialystok Poland. Written informed consent to participate in this study was provided by the participants' legal guardian/next of kin.

## Author Contributions

MK, AP, and EM: conception and design, acquisition of data, analysis and interpretation of data, and drafting of the manuscript. AH: acquisition of data, analysis and interpretation of data. WD: revising of the manuscript. All authors read and approved the final manuscript and certify that the manuscript is a unique submission and is not being considered for publication by any other source in any medium. The manuscript has not been published, in part or in full, in any form.

## Conflict of Interest

The authors declare that the research was conducted in the absence of any commercial or financial relationships that could be construed as a potential conflict of interest.

## Abbreviations

CDU: Color-Doppler Ultrasound.
